# Design of 2D Sparse Array Transducers for Anomaly Detection in Medical Phantoms

**DOI:** 10.3390/s20185370

**Published:** 2020-09-19

**Authors:** Xiaotong Li, Anthony Gachagan, Paul Murray

**Affiliations:** Department of Electronic and Electrical Engineering, University of Strathclyde, Glasgow G1 1XW, UK; a.gachagan@strath.ac.uk (A.G.); paul.murray@strath.ac.uk (P.M.)

**Keywords:** sparse array, ultrasonic transducer, particle detection

## Abstract

Aperiodic sparse 2D ultrasonic array configurations, including random array, log spiral array, and sunflower array, have been considered for their potential as conformable transducers able to image within a focal range of 30–80 mm, at an operating frequency of 2 MHz. Optimisation of the imaging performance of potential array patterns has been undertaken based on their simulated far field directivity functions. Two evaluation criteria, peak sidelobe level (PSL) and integrated sidelobe ratio (ISLR), are used to access the performance of each array configuration. Subsequently, a log spiral array pattern with −19.33 dB PSL and 2.71 dB ISLR has been selected as the overall optimal design. Two prototype transducers with the selected log spiral array pattern have been fabricated and characterised, one using a fibre composite element composite array transducer (CECAT) structure, the other using a conventional 1–3 composite (C1–3) structure. The CECAT device demonstrates improved coupling coefficient (0.64 to 0.59), reduced mechanical cross-talk between neighbouring array elements (by 10 dB) and improved operational bandwidth (by 16.5%), while the C1–3 device performs better in terms of sensitivity (~50%). Image processing algorithms, such as Hough transform and morphological opening, have been implemented to automatically detect and dimension particles located within a fluid-filled tube structure, in a variety of experimental scenarios, including bespoke phantoms using tissue mimicking material. Experiments using the fabricated CECAT log spiral 2D array transducer demonstrated that this algorithmic approach was able to detect the walls of the tube structure and stationary anomalies within the tube with a precision of ~0.1 mm.

## 1. Introduction

Transcranial ultrasonography has become an important method for stroke diagnosis, as it is a non-invasive, low-cost, and safe test. Compared to other stroke diagnosis techniques, such as computed tomography (CT) and magnetic resonance imaging (MRI), transcranial ultrasound is more comfortable and suitable for most patients as no noise or radiation is generated during the test. The main disadvantage of transcranial ultrasound is its operator dependency [1]. Different from CT and MRI, transcranial ultrasound cannot provide an image of the brain but only information associated with blood flow situation. To identify the blood vessels, the operator must adjust the position of the probe to obtain signals from different directions throughout the test. Thus, the experience of the operator could highly affect the diagnostic efficiency and has limited the application of transcranial ultrasound in situations when experienced ultrasound operators are not available. This provided the motivation for the work described in this paper, with two technical aspects considered to reduce operator dependency:

1. Design a 2D array transducer to achieve enhanced and flexible imaging capability.
2. Develop an image processing approach to automatically detect anomalies in the blood flow.

The speed of sound in human tissue is assumed to be 1540 m/s [2]. Hence, for an operating frequency of 2 MHz, which is the working frequency for most transcranial ultrasound transducers [3], the wavelength is 0.77 mm. Due to the associated half-wavelength criteria for element pitch in dense imaging array designs [4], a full matrix 2D array transducer would require ~5000 array elements for a 30 mm diameter active area. This presents a significant challenge for both manufacturing and controlling the array. The approach using a 2D sparse array transducer, if designed correctly, can achieve useful imaging capability.

According to the element distribution, the sparse array can be further divided into two groups, one is the periodic sparse array and the other is the aperiodic sparse array. The design process of the periodic sparse array is relatively straightforward. It mainly focuses on increasing the element pitch of the conventional dense array. However, the sidelobe and grating lobe level of the periodic sparse array can be relatively high [4]. Compared to the periodic sparse array, the aperiodic sparse array can avoid the formation of grating lobes since the aperiodic configuration reduces the interaction between elements [4]. Optimization algorithms, such as simulated annealing (SA), genetic algorithm (GA) and minimum redundancy linear array [5,6,7], have been investigated in sparse array design. Generally, these methods intend to find the set of elements that can achieve a desired beam pattern from a large amount of periodically arranged grids [8]. An approach using SA and non-gridded elements proposed in [9] and further developed to be combined with density tapering [10] has achieved improved results but employs complex algorithms and could be time consuming to design.

**Figure 1:**
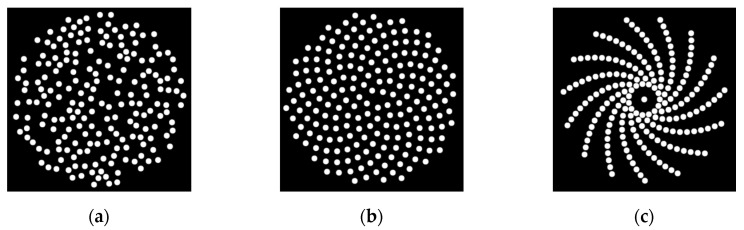
Three sparse array configurations which have been simulated in this work: (**a**) random array; (**b**) sunflower spiral array; (**c**) log spiral array.

An interesting element pattern that has been studied for sparse array design is the spiral pattern. The spiral pattern avoids the periodic structure that forms the grating lobes. At the same time, it allows for more control over the position of each element [4]. Two types of spirals which have been well studied for sparse array design are the Fermat and the logarithmic spirals, with examples illustrated in Figure 1b and Figure 1c, respectively. The Fermat spiral has been used in designing antennas [11] and ultrasound sparse array transducers [8,12,13]. Combined with density tapering, these designs can achieve low sidelobe levels while maintaining acceptable sensitivity. For the Fermat spiral, there is a special case when the step angle is set to 137.51° [12], which, due to its shape, is also called the sunflower spiral [12], as illustrated in Figure 1b. The logarithmic spiral was used to design a 2D sparse array used in non-destructive evaluation (NDE) [4]. As a fractal structure, the logarithmic spiral benefits from its self-similarity property, which spreads the sidelobe energy [4].

Typically, a 1–3 connectivity piezoelectric composite configuration would be used as the active material for operation into a low acoustic impedance load media, such as tissue [14]. However, the aperiodic sparse array configuration of interest does not align well with the regular pillar arrangement within a standard 1–3 material. Hence, a flexible piezoelectric ceramic structure, called the composite element composite array transducer (CECAT), is used as the basis for the developed 2D array configuration. The CECAT configuration was proposed in [15] to increase the flexibility of a piezoceramic-based ultrasonic transducer. Elements in the CECAT configuration are individual piezocomposites consisting of piezoelectric fibre material embedded in a polymer phase [15]. The piezoelectric fibres are randomly placed within the array element area and the experimental results demonstrate that this random fibre structure can achieve similar performance when compared with the conventional 1–3 composite structure [15]. Moreover, the random configuration can suppress the inter-pillar mode since it breaks the periodic pillar arrangement in the matrix configuration.

Image processing is another aspect that could improve the operator dependency of the transcranial ultrasound. An ultrasound imaging technique, called transcranial colour-coded duplex ultrasonography (TCCS), has been developed to provided 2D colour-coded Doppler images using 1D ultrasound array, which contain information associated with the blood flow direction and velocity, for intracranial vessels [16]. However, as this is a 2D imaging technique, the operator still needs to manually adjust the position of the probe during the test [3]. Researchers have also studied the feasibility of 3D transcranial ultrasonography. In [17], a helmet is produced to hold two 2D sparse phased array transducers at opposite sides of the transtemporal windows to achieve 3D imaging of the intracranial vessels. Contrast agents and aberration corrections have been studied to improve the quality of 3D images [18,19]. In this work, in addition to developing an efficient 2D transducer design, an image processing algorithm, which utilises the Hough transform, image subtraction and morphological opening, is proposed for the automated detection of vascular walls and anomalies in the blood flow. Standard laboratory instrumentation and tissue mimicking materials, which represent a simple vascular scenario in specific phantom configurations, are used to develop and evaluate the image processing algorithm.

## 2. Design of Sparse Array Pattern

### 2.1. Sparse Array Configurations

The array transducer aperture size was set to match the size of the temporal window, with a 30 mm diameter chosen for the current design [19]. Circular elements were utilised to achieve rotational symmetry. Three aperiodic array configurations were simulated to find an optimised design: the random array, the logarithmic spiral array, and the sunflower spiral array. Figure 1 illustrates examples of these array configurations. In this work, the position of an element is determined by the coordinate of its centre point. Thus, the radius of the aperture ($R_{ap}\;$) within which to place the centres of the elements would be less than 15 mm and can be expressed as

(1) $$R_{ap}=R_{org}-ele\_r$$

where $R_{org}=15\;mm$ and ele_r is the radius of the array element.

#### 2.1.1. Random Array Pattern

The position of the elements in the random array were arbitrarily selected within the aperture. To avoid overlapping between array elements, a minimum distance between two elements was set, which can be expressed as

(2) ele_pitch =2∗ele_r+ele_g

where ele_g is the minimum gap between two elements.

Since the aperture size is fixed in this work, there is an upper limit ($n_{max}\;$) to the number of elements (ele_num ) for the random array configuration. In the design process, ele_num_max is defined as the maximum number of sub-squares that can be placed inside the maximum internal square of the aperture. The side length of the sub-squares is equal to the pitch. This definition can be expressed as

(3) $$n_{\max}=\frac{{\left(2R_{ap}\right)}^{2}}{ele\_pitch^{2}}$$

While alternative methods could have been used, such as hexagonal packing, this approach was preferred to ensure the stability of the process.

#### 2.1.2. Sunflower Spiral Array Pattern

As previously mentioned, the sunflower spiral array is a special case of the Fermat spiral array. The mathematical form of the sunflower array is

(4) $$r_{n}=a_{sun}\sqrt{\gamma_{n}}=a_{sun}\sqrt{n\beta },\;\;\beta =137.51{}^{\circ}\;\left(2.4\mathrm{rad}\right)$$

where n is the *n*th point in the sunflower spiral array, $r_{n}$ is the radial distance of the *n*th point, and γ is the polar angle. $a_{sun}$ is a constant parameter relative to the shape of the sunflower spiral array [12]. It can also be written as

(5) $$x_{n}=r_{n}*\cos\left(n\beta \right),\;y_{n}=r_{n}*\sin\left(n\beta \right)$$

where $x_{n}$ and $y_{n}$ are the rectangular coordinates of point n.

The process to generate a sunflower spiral array starts from generating a set of points which distribute along the sunflower spiral. These points are called initial points. To ensure that all initial points generated are within the desired circular aperture, there is a limitation, which can be expressed as

(6) $$r_{last}=a_{sun}\sqrt{\gamma_{last}}=a_{sun}\sqrt{n_{last}*\beta }\leq R_{ap}$$

where $r_{last}$ and $\gamma_{last}$ are the radial distance and the polar angle of the last point, $n_{last}\;$. According to Equation (6), $a_{sun}$ and $n_{last}$ are dependent on each other. Thus, there are two methods to generate the initial points. In this paper, we set $n_{last}$ first, with both options explored in detail in [20]. Importantly, further analysis proves that the maximum number of elements ($n_{max}$) that can be placed within the aperture area is independent of $a_{sun}$ and $n_{last}$. Hence, as long as ele_pitch remains unchanged, both methods provide the same $n_{max}$ values, which can be expressed as

(7) $$n_{max}\leq \lfloor \frac{2.48R_{\mathrm{ap}}{}^{2}}{\beta *ele\_pitch^{2}}\rfloor$$

#### 2.1.3. Log Spiral Array Pattern

A logarithmic spiral array consists of one or several log spiral lines (arms), as illustrated in Figure 1c, and can be expressed with the following polar equation:

(8) $$r=a_{log}*e^{b\left(\gamma +\theta \right)}$$

where $a_{log}$ and b are constant parameters that affect the shape of the spiral. $a_{log}$ determines the minimum distance from the origin to an arm, while b controls the curvature of each spiral arm. θ is the rotational angle between two adjacent spirals. To ensure that no arms overlap with each other, the parameters $a_{log}$ and b need to be adjusted. First, the minimum distance between the first elements of two adjacent arms must be larger than the diameter of the element. This condition can be expressed as

(9) $$2a_{log}*sin\left(\frac{\theta }{2}\right)>2ele\_r$$

In this work, the minimum gap between the first elements of two adjacent arms is set to equal half of the gap between adjacent elements along the same arm.

Importantly, once the parameters are fixed, the layout of the log spiral array is set.

### 2.2. Peak Sidelobe Level (PSL) and Integrated Sidelobe Ratio (ISLR)

Two metrics, known as peak sidelobe level (PSL) and integrated sidelobe ratio (ISLR), are used to estimate the performance of the array configurations in terms of their imaging capability [4,21]. These two parameters reflect the relative relationship between the main lobe value the associated sidelobe. PSL is defined as the ratio of the maximum sidelobe value to the maximum main lobe value. ISLR is defined as the ratio of the total energy contained outside the main lobe (sidelobe area) to the energy contained inside the main lobe. These two parameters can be expressed using

$$PSL=20\log_{10}\left(A_{s}/A_{m}\right)$$

(10) $$\;ISLR=10\log_{10}\left(E_{s}/E_{m}\right)\;$$

where A and E represent the amplitude and the energy in the lobe(s), respectively. The sidelobe is represented by subscript s , while the main lobe is represented by subscript m . In this work, the threshold between the main lobe and the sidelobe is set to be −10 dB.

Both PSL and ISLR are related to the quality of the image which could be achieved by an ultrasonic transducer. They reflect the ability of the imaging system to identify the target in the main lobe path in the presence of targets of scatters in the sidelobe region. High PSL or ISLR values could indicate a problem when the target in the main lobe path is weak [21] or when imaging low contrast materials [4]. Thus, an appropriate transducer should have a low PSL value as well as low ISLR.

To reduce the computational time, in this work, a 2D fast Fourier transform (FFT) was used to predict the propagation of ultrasound waves in the far-field area by applying the 2D FFT on the aperture function of the array [22].

### 2.3. Simulation Process and Results

The three sparse array configurations shown in Figure 1 were first modelled in MATLAB using a 2D FFT to predict the directivity functions in the u-v space [23]. As explained in Section 2.1, for each configuration, a different number of parameters was required to design the array aperture. A set of candidate values were created for each parameter. For the purpose of comparison, the parameters common to all array configurations were set to have the same candidate values for all the array configurations. Table 1 lists the parameters and corresponding candidate value intervals.

The parameter ele_r, which is the array element radius, needs to be modified by all three array configurations. For 1–3 composite transducer, there should be at least 9 (3 × 3) elements under each electrode [24]. However, the PZT fibres (Smart Material Corp., Sarasota, FL) used in this project could only reach 75% of the properties for bulk PZT materials [25]. Thus, with 50% volume fraction and 250 µm diameter PZT fibres, the minimum element radius is set to be 0.7 mm. The parameter ele_g is the minimum gap between two elements. However, for different array configurations, the definition of ele_g is slightly different. For random array and sunflower spiral array, ele_g is the minimum distance between two elements within the same array. For log spiral array, ele_g is the gap between two adjacent elements along the same arm. As the transducer is planned to be manufactured as a CECAT, to prevent fibres from neighbouring elements from crossing over to each other, the lower limit of ele_g is set to 0.2 mm for random array and sunflower array and 0.4 mm for log spiral array. These numbers were determined based on a CECAT manufacturing trial. The minimum distance between elements in the log spiral array is affected by several variables. Thus, the minimum ele_g for the log spiral is set as a larger value in order to prevent fibres in neighbouring elements from crossing. ele_r and ele_g affect the maximum number of elements that can be placed within the fixed aperture, and it should be noted that a large ele_g will negatively influence the sensitivity of the transducer. Thus, ele_r and ele_g must be constrained and the upper limits for ele_r and ele_g are chosen to be 1.5 mm and 1 mm to align with practical transducer fabrication logistics.

**Figure 2:**
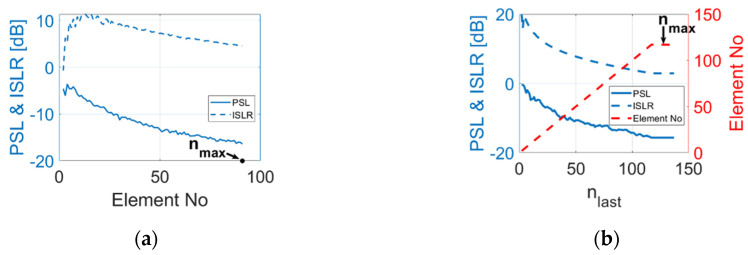
PSL and ISLR performance of (**a**) random array configuration and (**b**) sunflower array. configuration with respect to number of elements within the array.

For the random array and the sunflower spiral array, the maximum number of elements varies with the settings of both ele_r and ele_g . Therefore, for these two configurations, there is no requirement to introduce new parameters based on the number of elements. Simulations were processed to find the relationship between element number and PSL as well as ISLR. The results are shown in Figure 2. For the random array, 50 samples were simulated for each element number, and the minimum values for PSL and ISLR are presented in Figure 2a. As illustrated in the figures, for both random array and sunflower array, PSL and ISLR tend to decrease with the increase in element numbers. Thus, for random array and sunflower array, the number of elements within an array is set to equal the upper limit of the element number and only ele_r and ele_g are variable in the optimisation process.

By iterating through all candidate parameter values for each configuration, all possible sparse array patterns have been simulated, with the corresponding PSL and ISLR calculated and stored. The minimum PSL is −17.85 dB for the random array, −17.43 dB for the sunflower array and −20.29 dB for the log spiral array. Thus, in terms of PSL, the log spiral array performs better than the other two array configurations.

**Figure 3:**
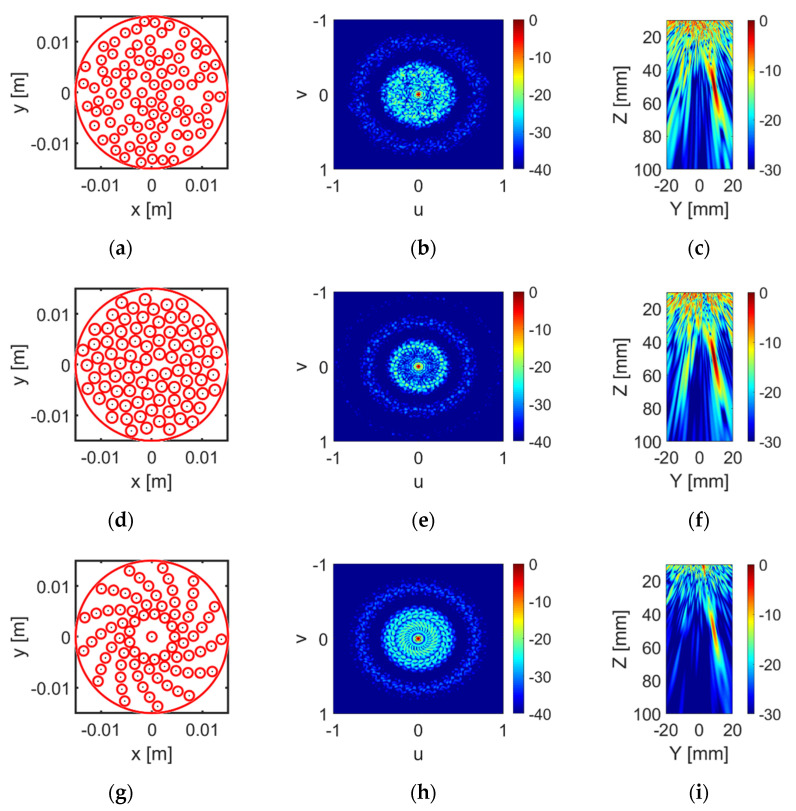
(**a**,**d**,**g**) illustrate the layout of the optimised random array, sunflower array and log spiral array separately. The corresponding unsteered directivity functions are illustrated in (**b**,**e**,**h**). (**c**,**f**,**i**) show the steered beam profile (10°) for each array configuration.

However, further analysis indicates that the configuration with the lowest PLS has relatively higher ISLR compared with other configurations with similar PSL. Thus, instead of selecting the layout with the lowest PSL, a configuration with low PSL and low ISLR was selected for each configuration. Other factors, such as the manufacturing complexity and practical realization feasibility, were taken into account. Figure 3 illustrates the selected array pattern for each array pattern, as well as the corresponding unsteered and steered (focused at 50 mm with 10° steer angle) directivity functions. The PSL and ISLR values of the selected pattern for each array configuration are shown in Table 2. As shown in the table, the log spiral array has the lowest PSL, while the sunflower spiral array performs the best in terms of ISLR. Since the elements in the log spiral array are placed more regularly than in the sunflower array, it is easier to fabricate a CECAT with a log spiral layout. Thus, the log spiral array pattern was selected as the final design for this prototype array. There are 13 arms, with 6 elements within each arm and an extra element in the centre of the array, i.e., 79 elements in total. The parameters used to define this log spiral array pattern were $a_{log}\;$: 4.5 mm; b: 1.2; and θ: 27.69 deg. The element radius is 0.95 mm and the gap between adjacent elements along the same arm is 0.5 mm.

## 3. Transducer Manufacturing and Characterisation

### 3.1. Fabrication of Prototype Transducers

**Figure 4:**
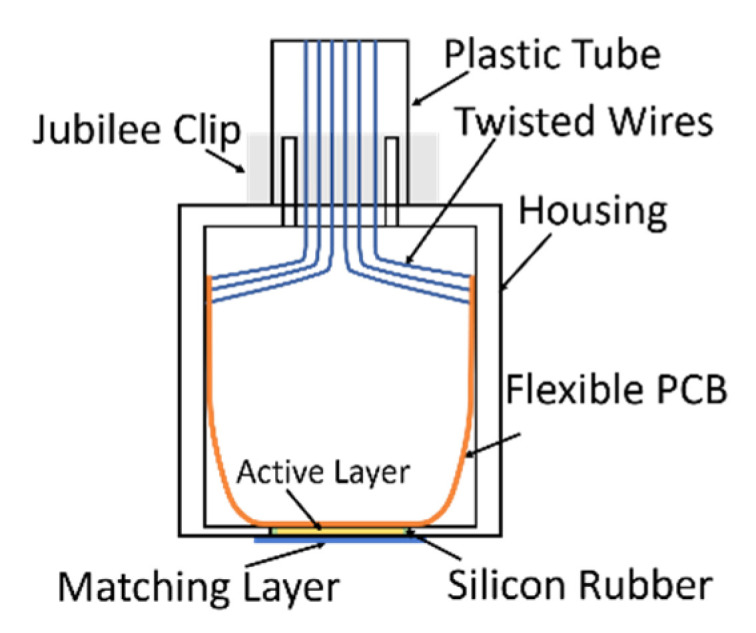
Illustration of prototype transducer structure.

A pair of prototype transducers with the 79-element log spiral array pattern were manufactured, one using a fibre CECAT active layer and the other incorporating a conventional 1–3 composite (C1–3) active layer. Both array devices will have the same electrode pattern but will have different piezoelectric microstructures. These two transducers will enable comparison between the fabrication processes in terms of acoustic performance of the log spiral array design. Figure 4 illustrates the construction of the prototype transducers.

**Figure 5:**
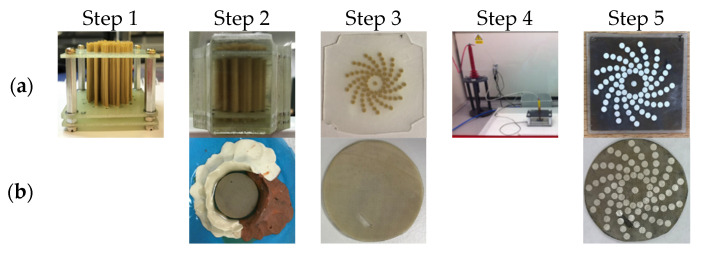
Illustration of the manufacturing process for (**a**) the CECAT active layer and (**b**) the C1–3 active layer.

The key difference between the fibre CECAT and the C1–3 transducers is the active layer manufacturing process, as illustrated in Figure 5, and corresponding piezoelectric microstructure. The following description of the CECAT fabrication process relates to the process stages illustrated in Figure 5a. (1) For the CECAT active layer, a jig was made to hold the fibres in the desired positions, corresponding to the array element locations illustrated in Figure 3g, to facilitate fabrication. The volume fraction of each individual active array element was calculated to be 50%, which ensured good piezoelectric performance and manufacturability of the device [15]. Then, 250 μm PZT5A fibres (Smart Material Corp., Sarasota, FL, USA) were selected as the piezoelectric material. Therefore, each individual array element, which has a 0.95 mm radius, contains 28 individual fibres. (2) The jig with the fibres was placed inside a rectangular mould and CIBA-GEIGY CY221-HY956 epoxy (medium set) poured into the mould and left to cure. This medium set material was selected from the CUE materials database as it provided a compromise between low longitudinal attenuation and high shear attenuation whilst, importantly, being suitable for machining [26]. (3) After curing processing, the composite was then machined to 0.75 mm thickness to achieve the 2 MHz operating frequency. (4) Before applying the log spiral electrode pattern, the fibre CECAT active layer was fully electroded using silver paint (AGG302, Agar Scientific Ltd., Essex, UK) and poled. The fibre CECAT active layer was polarised at 1.5 kV for 15 min, at room temperature, in accordance with the PZT fibre manufacturer’s guidelines [25]. (5) Finally, the silver paint was removed from the material and array electrodes were added to one face of the poled CECAT composite slice using metallic evaporation through a mask representing the log spiral array layout—see Figure 3g—while the ground electrode was applied by fully covering the other face of the slice through the same evaporation process. Silver (Ag) was used as the material for electrodes (~500 nm), with a thin layer (~20 nm) of chrome (Cr) evaporated first to improve adhesion to the active material.

For the C1–3 active layer, the standard “dice and fill” method [27] was used. To maintain consistency between the two array devices, design parameters including the piezoelectric material, the epoxy resin, the volume fraction and the electrodes materials were kept constant during the fabrication. The final thickness of the composite layer was 0.78 mm, which is slightly thicker than the CECAT layer and will result in a downward shift in operating frequency for this device. The pillar width and the kerf width for the C1–3 active layer are set to 0.22 mm and 0.09 mm, respectively. This gives around 29 pillars within each array element, which is very close to that of the CECAT active layer (28 fibres per element). After the dicing process, the fabrication process matches stage 2, 3 and 5 associated with the CECAT production, as illustrated in Figure 5b.

The electrical connection between the active layer and the external power source is achieved through two steps, first bonding the active layer to a flexible PCB using anisotropic conductive epoxy (Creative Materials Inc., Ayer, MA, USA), which is only conductive in the thickness direction, and then soldering twisted-pair wires to the PCB. Twisted-pair wires are used to reduce noise between alternate signal channels within the array. The active layer, the flexible PCB and the twisted-pair wires connected to the PCB are packaged inside a metallic housing, as illustrated in Figure 5. A quarter wavelength matching layer made of 4% tungsten and CIBA-GERIGY CY1301-HY1300 (hard set epoxy), selected from the CUE materials database [26], was applied on the prototype transducers to reduce the mismatching of acoustic impedance between the active layer and the load material (tissue or water). Finally, the length of twisted-pair wires, for external connection, was packaged inside a length of plastic tubing. Silicon rubber and a 5-min two-part epoxy (Araldite Rapid, Huntsman Advanced Materials, Switzerland) were used to seal the transducer and make it waterproof for testing using the tank-tube phantom.

### 3.2. Impedance Response

**Figure 6:**
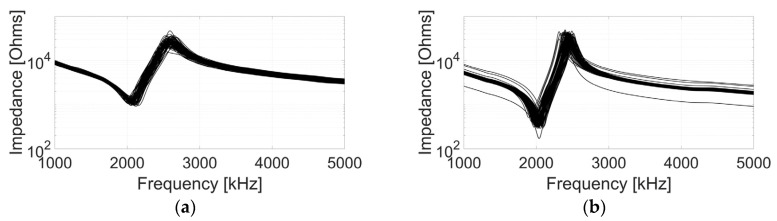
Impedance response of all array elements for (**a**) the CECAT and (**b**) the C1–3.

The electrical impedance response of all array elements was measured before bonding to the flexible PCB. The active layers were air loaded when carrying out the measurement to minimise damping to the array elements. The results are shown in Figure 6 and it is evident that, for each active layer, the individual electrical impedance characteristics vary within a limited range. Moreover, the C1–3 has lower electrical impedance, which indicates that, with the same driving voltage, the C1–3 would have higher vibration velocity, i.e., perform better in terms of the sensitivity.

Three transducer parameters—the resonance frequency ($f_{r}$), the anti-resonance frequency ($f_{a}$) and the coupling coefficient ($k_{t}$)—are used to evaluate the piezoelectric performance of the active layers and the individual array elements [28]. The averaged values for each parameter are listed in Table 3. As shown in the table, the CECAT and the C1–3 have similar $f_{r}$, while the CECAT has higher $f_{a}$ than the C1–3. This situation is associated with the better performance of the CECAT in terms of $k_{t}$ (0.64 to 0.59). The standard deviation results shown in Table 3 indicate that the CECAT has better uniformity in terms of $f_{r}$ and $f_{a}$, while the array elements in the C1–3 perform better in terms of $k_{t}$.

### 3.3. Inter-Element Cross-Talk

**Figure 7:**
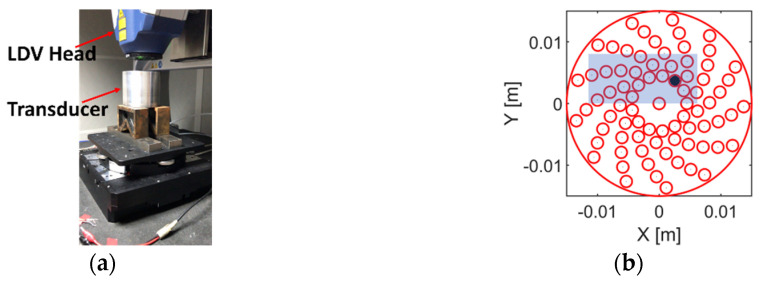
(**a**) Illustration of the experimental setup for mechanical cross-talk measurement. (**b**) Illustration of how the LDV scanning was processed. The black circle represents the fired array element. The blue block represents the area that was scanned.

The mechanical cross-talk between neighbouring array elements was measured using a 3D Laser Doppler Vibrometer (LDV) (Polytec Inc., Waldbronn, Germany). The measurement was conducted with the active layer, the PCB and the twisted wires packaged in the housing and the matching layer attached. The experimental setup is shown in Figure 7a. The transducer was placed on a platform with the front face of the transducer (i.e., the ground side of the active layer) in parallel to the laser source. The laser source was set to focus on the matching layer. Reflected laser signal was captured and analysed by the LDV to evaluate the vibrational properties of the active layer. For each scan, only one array element was fired. Recall from Section 2.3 the log spiral array pattern; there are 13 arms with 6 elements in each arm, plus 1 element in the centre. Thus, there are seven types of element neighbouring situations in which mechanical cross-coupling should be investigated. Aiming to minimise scanning time, as opposed to scanning the entire active layer, only a region covering the fired element and its neighbouring elements, as shown in Figure 7b, was scanned.

**Figure 8:**
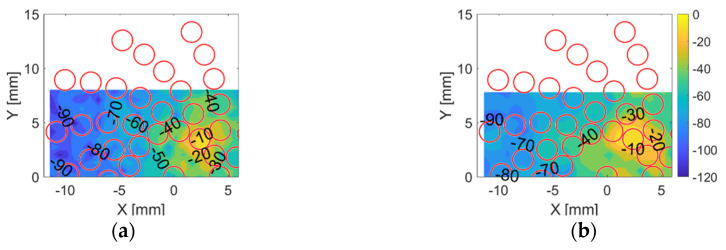
Cross-talk contours for (**a**) the CECAT and (**b**) the C1–3 when firing the first element in the 3rd arm individually. The red circles represent the theoretical position of the neighbouring elements.

Within each arm, the array elements are numbered 1 to 7 from the centre to edge. The cross-talk contours in dB for the first element within the 3rd arm in the CECAT and C1–3 are shown in Figure 8. This situation is presented here since the array elements are closer in the centre of the transducer than at the extremities and are thus of most interest for this experiment. As shown in Figure 8, the cross-talk is lower for the CECAT device as a result of the damping of the polymer phase between the array elements compared to the regular microstructure within the 1–3 device. However, the mechanical cross-talk is considered acceptably low for imaging applications in both devices.

### 3.4. Pulse-Echo Response

Characterisation of the transducer sensitivity (amplitude), bandwidth and pulse length were achieved using a pulse-echo test. A glass block was placed at the bottom of a tank, working as a reflector. The front surface of the transducer was positioned such that it was oriented in parallel to the glass block, with a separation distance of approximately 105 mm. The transducer was connected to a phased array controller, FIToolbox (Diagnostic Sonar Ltd., UK), and a 2 MHz half-cycle sine wave with 80 V amplitude was generated from the FIToolbox to drive each array element. The first received echo was recorded and analysed to extract performance metrics for each transducer.

**Figure 9:**
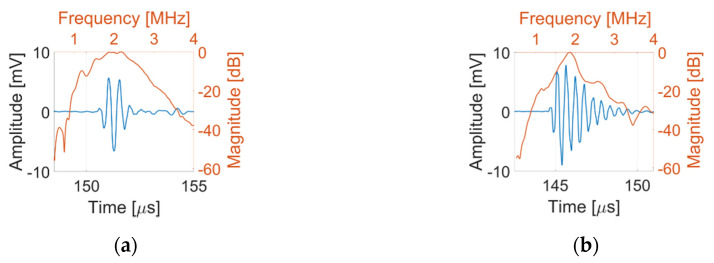
Pulse-echo response of the centre element for (**a**) the CECAT and (**b**) the C1–3.

Pulse-echo responses were successfully acquired from all 79 array elements from the C1–3, with three array elements from the CECAT failing to record a satisfactory response. Figure 9 presents example pulse-echo responses and corresponding spectra of the centre element from the CECAT and the C1–3. Table 4 lists the average values of central frequency, −20 dB pulse length, bandwidth and $V_{pp}$ for all the working array elements in each transducer. The centre frequency is 1.95 MHz for the CECAT and 1.85 MHz for the C1–3, both of which are slightly lower than 2 MHz desired working frequency. The averaged $V_{pp}$ for the CECAT is ~50% lower than the C1–3 measured performance. These results indicate that the CECAT has better spatial resolution, while the C1–3 performs better in terms of sensitivity.

## 4. Particle Detection Algorithm

In addition to the 2D sparse array transducer to provide more flexibility for transcranial imaging, an image processing algorithm that could automatically detect anomalies in the blood vessel is also of interest. A tank-tube phantom was made to represent a simple model of a blood vessel and consists of a 3.5 mm (inner diameter) plastic tube which is submersed in water. One end of the tube is placed underwater within a separate water tank, while the other end is connected to a syringe that is used to create a water flow through the tube. A 2.25 MHz, which is close to the desired 2 MHz working frequency in this project, 1D linear array transducer with 128 elements (Vermon, Tours, France) was used to image the tube-tank phantom. This transducer was used to provide array imaging capability to feed into the image processing development stage of the work, while the sparse 2D arrays discussed in Section 2 and Section 3 were being developed. The transducer was placed above the tube and in parallel with the bottom of the tank. Ball bearings in the range of 1–2 mm were placed at various positions inside the tube and within the area covered by the transducer aperture. The positions of the transducer and the tube remained fixed throughout the experimental process. The FIToolbox was used to control the transducer to collect data using the full matrix capture (FMC) method [29] at a sampling frequency of 50 MHz. The FMC data were then processed using the total focusing method (TFM) [29] to generate an image of the inspected area.

**Figure 10:**
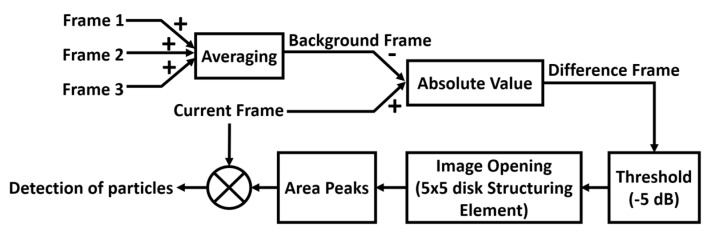
Block diagram to illustrate the particle detecting algorithm.

To simulate the concept of particles travelling through the imaging area, a series of TFM images starting with no particle and then with particles settled at different positions of the tube were also captured. During image processing, all TFM images were converted into grayscale images (frames) with pixel intensity varying between 0 and 1 (with 256 levels). In the grayscale images, pixels with an intensity equal to 1 are shown as white in the image, while pixels with an intensity equal to 0 are shown as black. The frame to be analysed is defined as the current frame, *CF*. An imaging algorithm, as illustrated in Figure 10, was used to locate the particles and estimate their size. The first step is to define a background frame, *BF*, which should represent the motionless objects in the image (i.e., the tube) using

(11) $$BF=\sum_{1}^{N}F\left(i\right)/N$$

where F(i) is the ith frame and N denotes the number of frames used in the calculation. In our experiments, N=3 as only three frames were used because the relative position between the transducer and the tube was kept the same during the imaging process, which means there will not be significant changes in the tube walls in adjacent frames. Thus, three frames would be a reasonable number considering both the accuracy of the resulted *BF* and the data processing time. In practice, especially for medical imaging systems that have more powerful data processing capability, more frames can be used to ensure the appropriate quality of the *BF*. The Hough transform [30] is applied to *BF* to detect the straight horizontal lines which represent the inner walls of the tube phantom. Then, a difference frame, *DF*, is computed by calculating the absolute difference of *BF* and *CF*. Ideally, when the object under inspection does not change, *DF* should produce an empty image (all pixels = 0). However, in practice, pixel intensities for the same inspected object can vary slightly through the sequence of frames, which leads to noise in *DF*. In our proposed image analysis pipeline, the noise is reduced by firstly applying a −5 dB threshold and then applying the morphological opening [31] with a 5 × 5 disk structuring element (SE). These parameters were empirically selected.

Particles are detected in the resulting noise-free *DF* by locating non-zero-pixel regions therein. Within each region, the pixel which coincides with the highest intensity pixel in the corresponding location in *CF* is recorded as the particle position. Since the particles settle at the bottom of the tube, the distance from each particle to the bottom inner wall of the tube is used to estimate its size. Furthermore, the distance from the particle to both inner walls is used to estimate the tube’s inner diameter.

**Figure 11:**
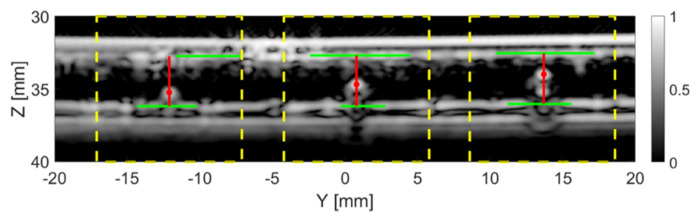
The current frame with the particles detected. The dashed yellow lines indicate the regions in which a particle was detected. The solid red lines represent the distance from the particles (red dots) to the top and bottom inner walls of the tube (solid green lines).

Figure 11 shows the results after applying the particle detection algorithm on the TFM images of the tank-tube from the 1D linear array. The true size of the particles in Figure 11, from left to right, is 1, 1.5 and 2 mm. All three particles were successfully located, with the results listed in Table 5. The estimated size is 0.95 mm for the 1 mm particle, 1.50 mm for the 1.5 mm particle and 2.05 mm for the 2 mm particle. The estimated inner diameter of the tube is 3.48 mm.

## 5. System Evaluation

### 5.1. Imaging Tube-Tank Phantom

The tank-tube phantom was used to test whether the CECAT could be used to detect anomalies in the tube and to estimate the size of the tube. The experimental setup is the same as described in Section 4, except that the 1D linear array was replaced with the CECAT. The particle detection algorithm which was developed in Section 4 was used to analyse the TFM images from the CECAT. The resolution of the TFM image from the CECAT is set to be 0.05 mm in both the *y*-axis and *z*-axis.

**Figure 12:**
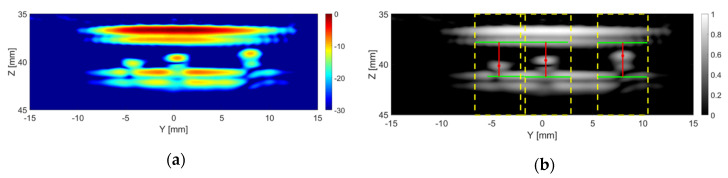
An example current frame from the CECAT with the particles detected: (**a**) is the TFM image and (**b**) presents the particle detection algorithm image result. The dashed yellow lines indicate the regions in which a particle was detected. The solid red lines represent the distance from the particles (red dots) to the top and bottom inner walls of the tube (solid green lines).

Figure 12a shows an example TFM image of the tank-tube phantom with three particles placed inside the tube, captured with the CECAT. All three particles can be clearly identified in the image. Figure 12b shows the results after applying the particle detection algorithm. All three particles were successfully located and dimensioned. The estimated size is 1.05 mm for the 1 mm particle, 1.65 mm for the 1.5 mm particle and 2.10 mm for the 2 mm particle. The estimated inner diameter of the tube is 3.42 mm.

### 5.2. Imaging TMM Phantom

To test the potential of the CECAT to be used in the biomedical field, phantoms which consist of tissue mimicking material (TMM) [32] were fabricated, where the TMM has appropriate scattering properties and acoustic properties that meet the International Electrotechnical Commission (IEC) standards [33].

**Figure 13:**
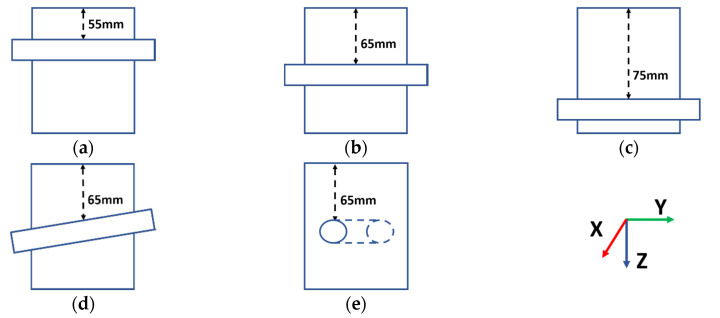
Illustrations of the basic structures of the lab phantoms. From (**a**–**e**), the phantoms are named the 55 mm phantom, the 65 mm phantom, the 75 mm phantom, the Angle V phantom and the Angle H phantom, respectively.

Figure 13 illustrates the basic structures of these lab phantoms. For the first three lab phantoms, as shown in Figure 13a–c, the tubes were placed in parallel with the *y*-axis at depths of 55, 65 and 75 mm from the top surface of the TMM, respectively. These distances were selected to match typical distances from the targeted blood vessels to the temporal window [34,35,36], with the tube aligned in parallel to the transducer front face. It should be noted that the tube used in these phantoms differs from the tank-tube phantom and has an inner diameter of 1/8 inch or 3.175 mm (Cole-Parmer UK, St Neots, Cambridgeshire). To broaden the scope of this experimental evaluation, two additional configurations, at a distance of 65 mm, were fabricated, with the tube located with a small angular orientation with respect to the configuration shown in Figure 13b. For the phantom shown in Figure 13d, the tube was firstly placed along the *y*-axis at 65 mm from the top surface of the TMM and then adjusted to have a 5° angle with respect to the *y*-axis in the Y-Z plane (i.e., vertical to the front surface of the transducer). For the phantom shown in Figure 13e, the tube was firstly placed along the *x*-axis at 65 mm from the top surface of the TMM, i.e., at right angles to the original configuration illustrated in Figure 13b. Moreover, the tube was also adjusted to have a 5° angle with respect to the *x*-axis in the X-Y plane (i.e., horizontal to the front surface of the transducer). These phantoms will be referred to as the 55 mm phantom, the 65 mm phantom, the 75 mm phantom, the Angle V phantom and the Angle H phantom, respectively.

**Figure 14:**
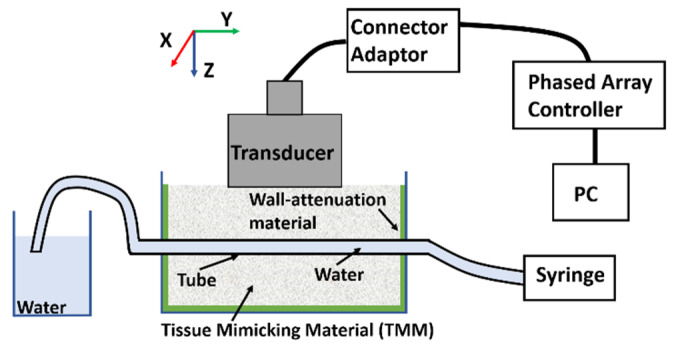
Illustration of the experimental setup for imaging the lab phantom using the CECAT.

**Figure 15:**
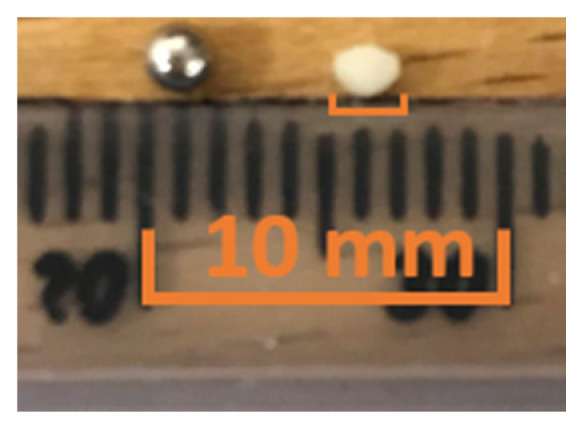
Illustration of the size and shape of the TMM particle. A 2 mm ball bearing is used as a reference.

Figure 14 illustrates the experimental setup for imaging the lab phantoms using the CECAT transducer. The setup is very similar to that of the tank-tube phantom. A syringe is used to fill water from a separate water tank into the tubes. The FMC method was used to collect data, while the TFM method was used to process images of the lab phantoms. A small piece of the TMM, as shown in Figure 15, was inserted into the tube to act as the particle artifact required to be detected. The TMM particle has an irregular shape as it was manually cut from a bulk of TMM and the property of the TMM makes it difficult to be machined into a regular small sphere. As shown in Figure 15, the edge length of the TMM particle varies between 1 and 2 mm. The TMM particle was firstly injected into the lab phantom using the syringe, as shown in Figure 14. Next, the CECAT was moved along the *y*-axis until the TMM particle could be seen from the TFM image. The CECAT was then fixed at that position and another three TFM images of the lab phantom were captured, with the TMM particle removed using the syringe. These three TFM images were used to process the background frame (*BF*) for the particle detection algorithm, as explained in Section 4.

**Figure 16:**
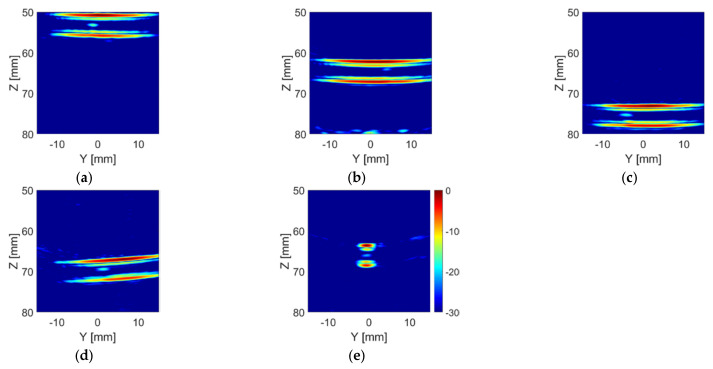
TFM image of (**a**) the 55 mm phantom, (**b**) the 65 mm phantom, (**c**) the 75 mm phantom, (**d**) the Angle V phantom and (**e**) the Angle H phantom with the TMM particle placed inside the tube from the CECAT.

**Figure 17:**
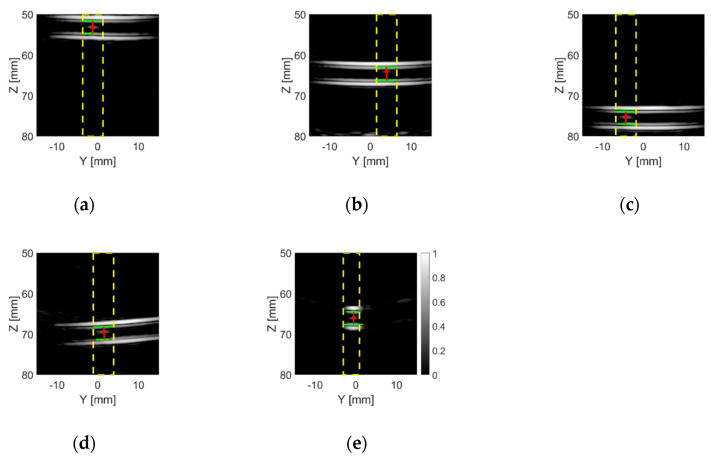
The results after applying the particle detection algorithm to the frames which were converted from the example TFM images of (**a**) the 55 mm phantom, (**b**) the 65 mm phantom, (**c**) the 75 mm phantom, (**d**) the Angle V phantom and (**e**) the Angle H phantom with the TMM particle placed inside the tube from the CECAT. The dashed yellow lines indicate the regions in which a particle was detected. The solid red lines represent the distance from the particles (red dots) to the top and bottom inner walls of the tube (solid green lines).

Figure 16 shows the TFM images of the phantoms with the TMM particle inside the tube. As expected, the intensity of the bright spots in the TFM images which represent the TMM particle is much lower than that of the ball bearings (as shown in Figure 12). This is because the acoustic impedance of the TMM particle is much closer to water than the steel ball bearing, which means that less sound energy will be reflected at the TMM–water interface than the steel–water interface. It can also be noticed that bright spots of the TMM particle have different intensities and shapes in different lab phantoms, as a consequence of the irregular shape of the TMM particle. These TFM images were analysed using the particle detection algorithm and the results are shown in Figure 17. For the Angle H phantom, the tube is located along the *x*-axis. Thus, the TFM image of the Angle H phantom in the Y-Z plane, as shown in Figure 16e, can only show a slice of the tube. This TFM image can still be analysed using the particle detection algorithm, as shown in Figure 17e, by zooming in to the area which contains the tube walls. The estimated TMM particle size and the estimated inner diameter of the tube for each TMM phantom are listed in Table 6, as well as the intensity for the pixel where the particle is located. The estimated particle sizes are all within the range of the TMM particle’s edge length. Moreover, the estimated tube inner diameters are all very close to the real tube size, which is 3.175 mm (1/8 inch).

## 6. Conclusions

This paper describes the design, fabrication and evaluation of a 2D sparse array ultrasonic transducer, whose design specification was developed for the potential to be applied for transcranial ultrasound imaging through the temporal window. Of the three sparse array configurations considered in this work, a log spiral array pattern was selected as a compromise between optimal PSL and ISLR values. Two prototype transducers, a piezoceramic fibre-based CECAT and a conventional 1–3 composite (C1–3), were manufactured to produce two similarly fabricated devices for comparison. Moreover, an image processing algorithm for detecting and sizing small particles was developed by applying the Hough transform, image subtraction and morphological opening to the computed total focusing method image from array transducers.

Characterisation results of the prototype transducers established that 4% of the array elements in the CECAT array were dead (null response in pulse-echo test). Interestingly, the CECAT structure demonstrated a reduced mechanical cross-talk level between neighbouring array elements (10 dB lower) and ~16.5% improvement in operational bandwidth when compared to the conventional C1–3 device. However, the C1–3 device produced a ~50% improvement in sensitivity from the pulse-echo experimental measurements.

Lab phantoms incorporating TMM with the tubes placed at different depths and orientations have shown that the CECAT can detect key features, which, in this case, is the tube walls and a TMM particle. Importantly, dimensions of the tube and the anomaly were accurately estimated (with 0.1 mm precision on average) by applying the developed image processing algorithms.

In summary, an ultrasonic array system was developed as a proof-of-concept design for transcranial ultrasound imaging application through the temporal window. The developed system comprises a 2D aperiodic ultrasonic array and signal/image processing algorithm. Unfortunately, at this stage, we were unable to compare our new CECAT array with a comparable commercial transducer. However, laboratory tests on targeted TMM phantoms demonstrated the potential of this technology for this application. Going forward, further modifications should be developed to improve the system performance, such as processing 3D images and evaluating the system with more representative medical phantoms. Moreover, for practical application, the next stage of the development cycle should limit the array aperture to 20 mm diameter to ensure compatibility across all patients and the particle sizing algorithm should be extended to measure anomalies without referencing the tube wall.

## Figures and Tables

**Table 1:** Parameters and candidate values for each array configuration.

Parameters	Interval	Array Type
Element radius (*ele___r*)	0.7:0.05:1.5 (mm)	All three array configurations
Minimum gap between two elements within the array (*ele___g*) * For the log spiral array, this would be the gap between adjacent elements along the same arm.	0.2:0.05:1 (mm)	Random and sunflower array
	0.4:0.05:1 (mm)	Log spiral array
Number of elements within each log spiral arm (*ele___per___arm*)	3:1:10	Log spiral array
Number of log spiral arms (*num___of___arm*)	7:2:19	Log spiral array
Constant parameter *b*	1.2:0.1:1.5	Log spiral array

**Table 2:** PSL and ISLR for each optimised pattern.

Array Type	PSL (dB)	ISLR (dB)
Random Array	−17.85	2.92
Sunflower Spiral Array	−17.38	0.58
Log Spiral Array	−19.33	2.71

**Table 3:** Analysed parameters for both active layers.

Array Type		CECAT	C1–3
$f_{r}$ **(kHz)**	Ave	2061	2030
	Std	33.6	39.4
$\;\;f_{a}$ (kHz)	Ave	2587	2440
	Std	43.5	44.5
$\;\;k_{t}\;\;$	Ave	0.64	0.59
	Std	0.014	0.007

**Table 4:** Pulse-echo response results for both prototype transducers.

Device	Centre Frequency (MHz)	Pulse Length (µs)	Bandwidth (%)	Peak-to-Peak Amplitude (mV)
CECAT	1.95	1.72	47.44	11.17
C1–3	1.85	2.67	30.95	16.67

**Table 5:** Location of the ball bearings in Figure 11.

Location	Ball Bearing Real Size		
	1 mm	1.5 mm	2 mm
Y (mm )	−12.10	0.80	13.70
Z (mm )	35.25	34.70	34.00

**Table 6:** Results of applying the particle estimation algorithms to TFM images shown in Figure 17.

Phantom Name	Estimated Particle Size (mm)	Estimated Inner Diameter (mm)	Particle Location Pixel Intensity (dB)
55 mm Phantom	1.64	3.16	−15.64
65 mm Phantom	2.15	3.20	−21.16
75 mm Phantom	1.79	3.09	−17.32
Angle V Phantom	1.93	3.16	−16.90
Angle H Phantom	1.65	3.15	−21.12
